# Dietary Fiber Estimate of DialBetesPlus App Users: Secondary Analysis of Data From a Randomized Controlled Trial

**DOI:** 10.2196/69340

**Published:** 2025-10-02

**Authors:** Wei Thing Sze, Kayo Waki, Ryohei Nakada, Toshimasa Yamauchi, Masaomi Nangaku, Kazuhiko Ohe

**Affiliations:** 1 Department of Clinical Information Engineering, Graduate School of Medicine, The University of Tokyo, 7-3-1 Hongo, Bunkyo-ku, Tokyo, 113-8655, Japan, 81 5800-9129; 2 Department of Planning, Information and Management, The University of Tokyo Hospital, Tokyo, Japan; 3 Department of Diabetes and Metabolic Diseases, Graduate School of Medicine, The University of Tokyo, Tokyo, Japan; 4 Division of Nephrology and Endocrinology, Graduate School of Medicine, The University of Tokyo, Tokyo, Japan

**Keywords:** type 2 diabetes, dietary fiber, mobile health, meal monitoring, diabetes self-management app

## Abstract

**Background:**

Despite the importance of dietary fiber in regulating glycemic control, the reported intake among patients with type 2 diabetes (T2D) in Japan was around 12‐16 g, well below the local official recommended intake of 20 g and above. Recent data is lacking, with the most recent available estimates collected between 2014 and 2019. Most mHealth dietary intervention apps for T2D focus on calorie and carbohydrate outcomes, with limited evidence on fiber intake. Fiber data was collected in a recent 12-month trial of DialBetesPlus, a multimodal diabetes mHealth self-management system that supports dietary behavior change by allowing users to record their meals and provides timely and detailed information on users’ nutrient intake.

**Objective:**

This study aimed to assess the pattern of dietary fiber intake among DialBetesPlus intervention users to provide recent data. As a secondary objective, the study explored factors that may influence dietary fiber intake among participants.

**Methods:**

Meal records were extracted using the DialBetesPlus app developer dashboard. Dietary fiber intake was measured for all intervention participants. The analysis included only data from participants with complete (breakfast, lunch, and dinner) meal records for at least 7 days. We calculated the average dietary fiber intake and fiber density per day by first averaging across all participants for each day, then averaging these daily values over 1 year. We averaged fiber intake per meal type across all days with available data for each participant, without excluding incomplete meal record days.

**Results:**

Out of 66 participants from the intervention group who were assigned to the DialBetesPlus intervention, 47 (71.2%) had at least 7 days of complete meal records and were included in the analysis. A 1-year trend analysis revealed a slight upward trend of daily fiber intake with the rolling average consistently below 18 g. The average fiber intake was 17.1 g/day, with a corresponding mean fiber density of 10.5 g/1000 kcal. The overall mean fiber intake was 17.1 g/day. Separate analysis by meal types revealed that the highest fiber intake was during dinner (6.7 g), followed by lunch (4.8 g), breakfast (4.4 g), and snacks (1.5 g), while fiber density was lowest for snacks (7.8 g/1000 kcal), followed by dinner (10.2 g/1000 kcal), lunch (10.5 g/1000 kcal), and breakfast (10.8 g/1000 kcal). No significant correlations were observed between average fiber intake and participant characteristics such as age, sex, BMI, hemoglobin A_1c_, blood pressure, and frequency of meal logging.

**Conclusions:**

Despite using a general diabetes self-management mHealth app (DiabetesPlus) that included dietary self-monitoring and basic nutritional feedback, users consumed less than the recommended 20 g/day of dietary fiber on average over a 1-year period. This study highlights the need to explore alternative mHealth strategies to further enhance dietary fiber intake among patients with T2D.

## Introduction

Dietary fiber is a macronutrient of importance in the dietary self-management of individuals with diabetes. Dietary fiber improves glycemic control by decreasing postprandial hyperglycemia, enhancing peripheral insulin sensitivity, and activating incretin secretion via short-chain fatty acids produced by fermentation of fiber in the intestines [1-3]. It also enhances satiety, which leads to a reduction in body weight [4].

Despite the importance of dietary fiber in regulating glycemic control, the reported dietary fiber intake among patients with type 2 diabetes (T2D) in Japan was around 12‐16 g, below the minimum intake of 20 g recommended by the Japan Diabetes Society [5-8]. Higher fiber intake (>25 g) is recommended for patients with T2D to prevent cardiovascular disease, reduce premature mortality, and to achieve meaningful hemoglobin A_1c_ (HbA_1c_) reduction [7,9]. Higher fiber intake was also reported to be associated with lower obesity risk and increased physical activity among patients with T2D in Japan [8].

Although many mHealth dietary self-monitoring apps target patients with T2D, most of them focus on calorie and carbohydrate intake, with limited evidence addressing fiber intake [10]. Furthermore, recent evidence on the fiber consumption of Japanese patients with T2D is limited, with the most recent available estimates collected between 2014 and 2019 [8]. Therefore, we performed a secondary analysis of the dietary data collected from a mHealth self-management support system that used the DialBetesPlus app to assess dietary fiber intake of the intervention participants. DialBetesPlus allows patients to self-monitor blood glucose, blood pressure, body weight, daily step counts, and diet. The effectiveness of DialBetesPlus on improving albuminuria and glycemic control in patients with diabetic kidney disease when added to standard care was demonstrated in a 12-month clinical trial conducted between 2018 and 2021 [11].

For diet self-monitoring, DialbetesPlus intervention participants recorded the content and quantity of their meals and snacks on the DialBetesPlus app via text input as well as registering a photograph of their meal to assist with the input. The recorded data was sent to the DialBetesPlus server and immediately evaluated using the FoodDataBank database (SARAH Co, Ltd). Based on the calculated consumed calories and nutrient intake, the server generated feedback related to nutritional balances and dietary habits, which were then sent to the patients through the app via text messages. DialBetesPlus strengthens perceived and actual control of dietary behavior modification by giving patients timely and detailed information about their nutrient intake, such as protein, fiber, and fat, as well as general tips and suggestions to eat healthier [11].

The aim of this secondary analysis is to explore patterns of dietary fiber intake of DialBetesPlus intervention users using meal records captured on the DialBetesPlus app, specifically on the overall average, trend over 1 year, and the average intake by meal type. As a secondary objective, we examined the association between total dietary fiber intake and participant characteristics such as age, sex, BMI, HbA_1c_, blood pressure as well as engagement parameters such as frequency of meal logging.

## Methods

### Overview

We conducted this secondary analysis using meal log data from a 12-month randomized controlled trial evaluating the effectiveness of DialBetesPlus, a self-management support system for diabetic kidney disease. The primary study randomized 132 participants from 8 clinical sites in Japan, 66 intervention and 60 control group between July 2018 and August 2019. Eligibility criteria included diagnosis of T2D with moderately increased albuminuria (urine albumin-creatinine ratio: 30‐299 mg/g creatinine), HbA_1c_ of 6.5% or more, and between 20 and 75 years of age [11]. We randomized participants to either an intervention group using DialBetesPlus, a self-management support system allowing patients to monitor exercise, blood glucose, diet, blood pressure, and body weight via a smartphone app or a control group receiving standard care.

**Figure 1.**
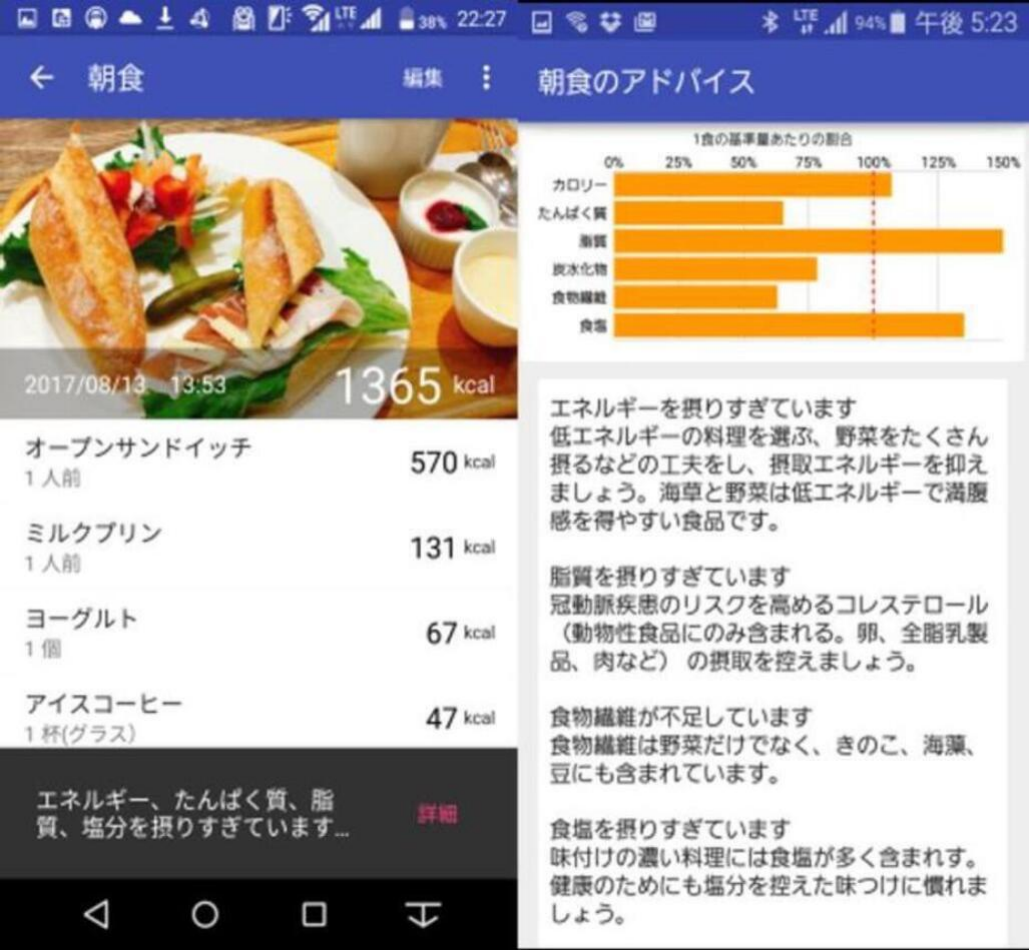
Example of nutrient assessment and feedback provided by the DialBetesPlus app after a meal log. English translation: left panel: meal type: breakfast; total energy: 1365 kcal; meal items: open sandwich – 570 kcal, milk pudding – 131 kcal, yogurt – 67 kcal, iced coffee – 47 kcal (1 glass). Note (bottom bar): "energy, protein, fat, carbohydrate, salt intake was excessive." Right Panel (breakfast advice): percentage of each nutrient relative to daily recommended intake: calories, protein, fat, carbohydrate, dietary fiber, and salt. For textual advice, please refer to Textbox 1.

After logging each meal in the DialBetesPlus app, participants received immediate feedback on the nutrient composition of the meal, including total energy (kcal), protein, fat, carbohydrate, dietary fiber, and salt content. For example, after logging breakfast, the app displayed the total caloric content of the meal along with a breakdown by individual food items. The app evaluated nutrient intake as a percentage of the recommended daily intake and provided personalized advice on each nutrient intake accordingly (Figure 1).

Textbox 1. **Textual Advice**

- You are consuming too much energy. Try to reduce your energy intake by choosing low-calorie dishes and eating lots of vegetables. Seaweed and vegetables are low in calories and help you feel full easily.
- You are consuming too much fat. Limit your intake of cholesterol (found only in animal-based foods such as eggs, full-fat dairy products, and meat), which increases the risk of arterial disease.
- You are consuming too little dietary fiber. Dietary fiber is found not only in vegetables, but also in mushrooms, seaweed, and beans.
- You are consuming too much salt. Heavily seasoned foods contain a lot of salt. For your health, try to get used to less salty foods.

We extracted both meal records and associated nutrient data including calorie, carbohydrate, fiber, salt content, and lipid from the DialBetesPlus app developer dashboard. We defined a complete meal record for a given patient and day as a record that had complete logged meal occasions for breakfast, lunch, and dinner in a day. Meal types (eg, “breakfast”) were selected by participants during the time of meal submission. We defined an incomplete meal record as one that was missing a meal record for any of breakfast, lunch, or dinner in a day, and days with an incomplete or missing meal log were excluded. To enhance the accuracy of dietary fiber intake estimates, we limited analysis to participants who recorded complete meals for at least 7 days, given that fiber intake is more accurately assessed from complete meal data.

### Statistical Analysis

We calculated the means and standard deviations for continuous variables, and frequencies and proportions for categorical variables, to summarize the baseline demographic and clinical characteristics of the study population. To assess whether there were any systematic differences between the analyzed and excluded groups at baseline, we conducted group comparisons using Welch *t* test or Mann-Whitney *U* test for continuous variables and chi-square test for categorical variables.

The average dietary fiber intake for each day was calculated by averaging the intake across all participants. To visualize the trend over time, a 30-day rolling average was applied to these daily averages. Then, we calculated the average fiber intake per day by taking the average of these daily values over 366 days. Similarly, we calculated fiber density (g/1000 kcal) each day by dividing each participant’s total fiber intake by their total energy intake, and then averaged the daily values across participants. We included days with at least 3 main meals (breakfast, lunch, and dinner) recorded in the analysis, regardless of whether snacks were logged. We took this approach because snack consumption tends to be more variable and inconsistent across individuals and days, making it a less reliable indicator of daily fiber intake.

To find mean dietary fiber intake for each meal type (breakfast, lunch, dinner, or snacks), the recorded dietary fiber intake for each participant was averaged per meal type across all days, and these averages were aggregated across all participants. Fiber density was also calculated for each meal type by dividing the fiber intake by the corresponding energy intake, standardized per 1000 kilocalories.

To explore the relationship between average fiber intake and individual characteristics, we calculated Spearman correlation. We explored the correlations between the average fiber intake per day and age, sex, baseline BMI, BMI change postintervention, baseline systolic blood pressure (SBP), baseline diastolic blood pressure (DBP), SBP and DBP change post intervention, baseline HbA_1c_, and HbA_1c_ change postintervention.

Python 3.12.4 (Python Software Foundation) was used for the data analysis.

### Ethical Considerations

The Institutional Review Board of the University of Tokyo School of Medicine approved this study (approval number 2021084NI-(2)). The approval includes permission for secondary analyses without requiring additional consent. We deidentified all study data to maintain confidentiality and protect the privacy of research participants. Participants in the original study received compensation as approved by the relevant ethics committee. No additional compensation was provided for this secondary analysis.

## Results

Out of 66 participants from the intervention group who used the DialBetesPlus app, 47 (71.2%) participants had at least 7 days with complete meal records and thus were included in the analysis (referred to as “analyzed participants” in this text). Participants were mostly male (72.3%, 34/47), late middle age or older individuals having a mean age of 60.2 (SD 8.6) years, with an average BMI of 28.1 (SD 5.3) kg/m^2^ (Table 1). Among the participants included in the analysis, the average percentage of days with complete meal records during the 1-year intervention period was 55.5% (203/366 days). There were no statistically significant differences in baseline characteristics such as age, sex, BMI, baseline blood pressure, and baseline HbA_1c_ between participants included in the analysis and those excluded (MultimediaAppendix 1  2).

**Table 1.** Demographic and clinical characteristics of the analyzed participants (N=47) in a 12-month randomized controlled trial investigating DialBetesPlus intervention for individuals with type 2 diabetes in Japan.

Demographic and clinical characteristics	Values (N=47)
Age (years), mean (SD)	60.2 (8.6)
Sex, n (%)	
Male	34 (72.3)
Female	13 (27.6)
Baseline BMI (kg/m^2^), mean (SD)	28.1 (5.3)
Changes in BMI (kg/m^2^) post intervention (n=46)	−0.7 (1.2)
Baseline HbA_1c_^a^, mean (SD)	7.8 (1.1)
Changes in HbA_1c_ post intervention	–0.2 (0.9)
Baseline blood pressure (SBP^b^/DBP^c^, mm Hg), mean (SD), (n=46)	135/81 (15/11)
Changes in blood pressure (SBP^b^/DBP^c^, mm Hg)	−3/−3 (18/13)

a HbA_1c_: hemoglobin A_1c_.

b SBP: systolic blood pressure.

c DBP: diastolic blood pressure.

**Figure 2.**
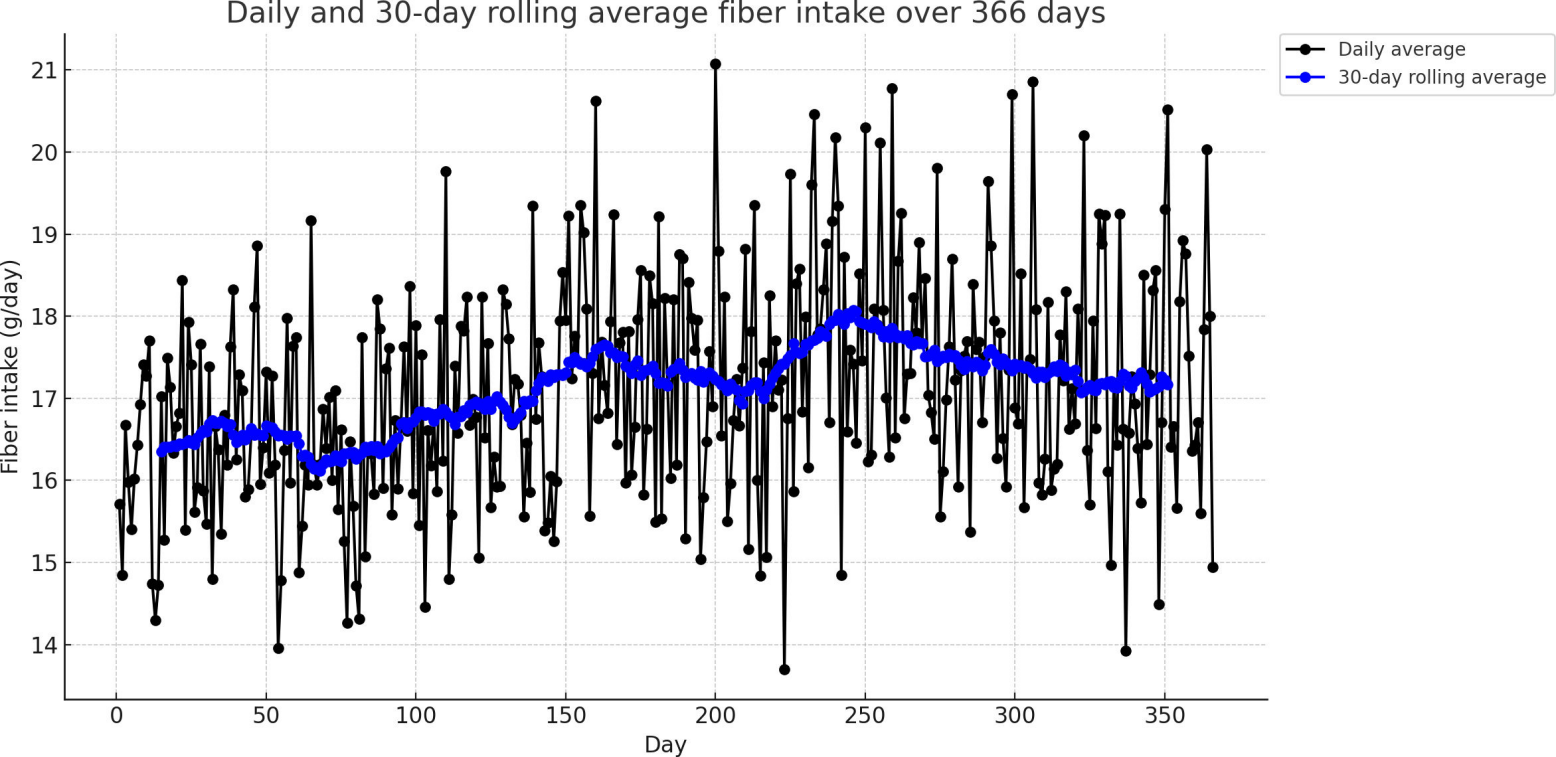
Thirty-day rolling average of daily dietary fiber intake over 366 days among analyzed participants (N=47) in a 12-month randomized controlled trial investigating DialBetesPlus intervention for individuals with type 2 diabetes in Japan.

**Figure 3.**
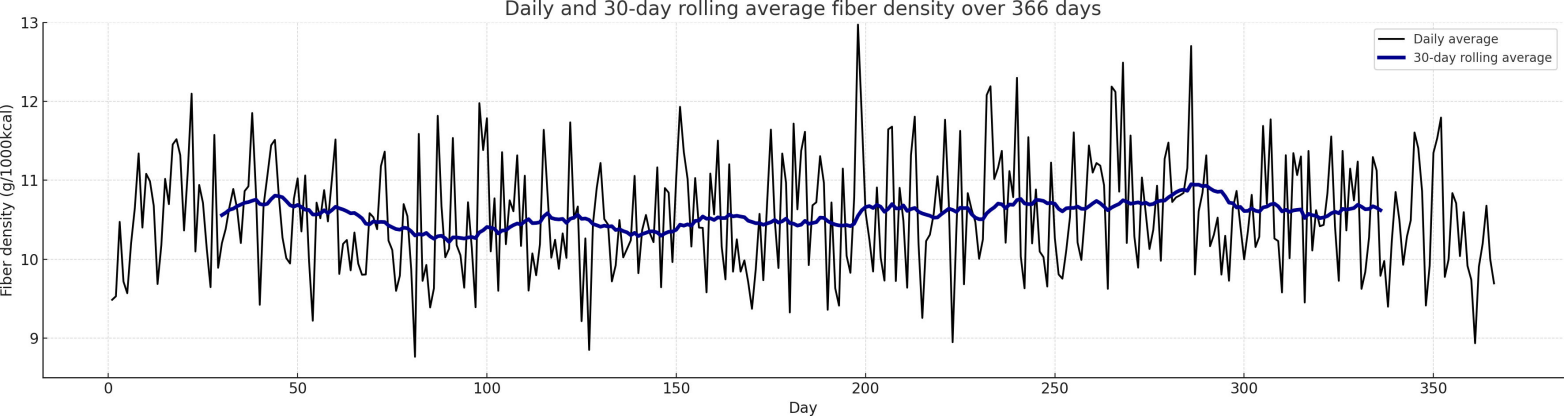
Thirty-day rolling average of fiber density over 366 days among analyzed participants (N=47) in a 12-month randomized controlled trial investigating DialBetesPlus intervention for individuals with type 2 diabetes in Japan.

Participants consumed an average of 17.1 (SD 1.4, SE 0.1) g/day of dietary fiber, with the 95% CI values ranging from 16.9 to 17.2 g/day. When adjusted for energy intake, the mean fiber density was 10.5 (SD 0.7, SE 0.04) g per 1000 kcal, with a 95% CI value of 10.5 to 10.6 g/1000 kcal. Visual inspection of Q-Q plots indicated that both daily fiber intake and fiber density were approximately normally distributed (Multimedia Appendix 3). The 12-month rolling average trend graph reveals that there was a gradual increase of approximately 1.5‐2 g over the first 6 months, followed by a slight decline of 0.5 g over the last 3 months, with the 30-day rolling average staying below 18 g most of the time (Figure 2). As for fiber density, the 30-day rolling average fiber density stayed relatively stable between 10 and 11 g/1000 kcal over the year with minor fluctuations (Figure 3). Analysis by meal types reveals that the highest fiber intake was during dinner (6.7 g), followed by lunch (5.8 g), breakfast (4.4 g), and snacks (1.5 g), while fiber density was lowest for snacks (7.8 g/1000 kcal), followed by dinner (10.2 g/1000 kcal), lunch (10.5 g/1000 kcal), and breakfast (10.8 g/1000 kcal; Table 2).

**Table 2.** Average dietary fiber and fiber density during breakfast, lunch, dinner, and snacks among analyzed participants (N=47) in a 12-month randomized controlled trial investigating DialBetesPlus intervention for individuals with type 2 diabetes in Japan. Absolute fiber intake (g).

Meal type	Mean (SD)		SE^a^ (95% CI)
Breakfast	4.3 (1.8)		0.3 (3.8-4.9)
Lunch	5.8 (1.9)		0.3 (5.3-6.4)
Dinner	6.7 (2.9)		0.4 (5.9-7.5)
Snacks	1.5 (0.7)		0.1 (1.3-1.7)
Fiber density (g/1000 kcal)			
Breakfast	10.8 (4.4)		0.6 (9.5-12.1)
Lunch	10.5 (3.6)		0.5 (9.4-11.5)
Dinner	10.2 (3.5)		0.5 (9.2-11.2)
Snacks	7.8 (4.5)		0.6 (6.5-9.2)

a SE: standard error.

Spearman correlation analysis revealed no statistically significant associations between the average fiber intake per day and participant characteristics including age, sex, baseline BMI, baseline HbA_1c_, baseline SBP, and baseline DBP, as well as changes in BMI, HbA_1c_, and blood pressure post intervention (all *P*>.05) (Multimedia Appendix 4). In addition, Spearman correlation analysis was conducted to examine the relationship between logging frequency and average daily fiber intake. Meal logging frequency was defined as the total number of meal logs logged over the study period. The analysis revealed no significant association between meal logging frequency and fiber intake (*P*=.03 and .82, respectively), suggesting that the meal logging frequency was not meaningfully related to the participants’ dietary fiber consumption.

## Discussion

### Principal Findings

In this secondary analysis, average dietary fiber intake among DialBetesPlus users was 17.1 g/day. Fiber intake gradually increased during the first 6 months of the intervention before declining slightly in the final 3 months. Fiber intake was highest at dinner and lowest from snacks. No significant associations were observed between total fiber intake and participant characteristics, including age, sex, baseline BMI, baseline HbA_1c_, or baseline blood pressure. Similarly, meal logging frequency showed no meaningful relationship with average fiber intake.

Our secondary analysis demonstrated that, even among this group of highly compliant and engaged app users, the average dietary fiber intake remained below the clinical recommendation of the Japan Diabetes Society, and the average fiber density fell well short of the general recommendation of 14 g/1000 kcal [12,13]. The DialBetesPlus app users performed diet self-monitoring and received feedback on nutrient intake and general dietary advice on the app over 1 year. These findings indicate that, despite the use of a sophisticated tool and high participant motivation, meeting fiber intake goals remains difficult, suggesting the need for more tailored or intensive support. Furthermore, patients might not be fully aware of the importance of eating more fiber or how to incorporate more fiber in their diet. Previous studies showed that various factors influence fiber intake, such as lack of knowledge on the benefits of fiber, beliefs on high-fiber food as unpalatable, the higher price of fiber-rich food, and lack of skills in recognizing sources of dietary fiber [14,15].

The average fiber intake per day of DialBetesPlus intervention users was generally higher than what was reported among the Japanese population with T2D in non-mHealth studies (17.1 g vs 12‐16 g) [5-7]. The trend graph shows a small and gradual increase of mean dietary fiber intake over the first 6 months (1.5‐2 g), which may be due to the influence of the intervention to help patients eat a healthier diet. The observed plateau in the fiber increase after 6 months is consistent with findings from previous studies showing that the effect size of mHealth intervention typically diminishes over time, possibly due to declining user engagement [11,16].

Breakfast was reported to contain the lowest fiber intake compared to lunch and dinner. Analysis of diet quality through Nutrient-Rich Food Index 9.3 showed that, on average, the percentage contribution of key nutrients (protein, dietary fiber, vitamins, and minerals) to the total dietary intake of Japanese was highest at dinner, followed by lunch, breakfast, and snacks [17]. The lower fiber intake observed at breakfast compared to lunch and dinner may be attributed to the simpler composition of morning meals, such as rice, plain bread, and miso soup, while lunch and dinner often featured a wider variety of vegetable side dishes [18]. Nevertheless, fiber density at breakfast was higher than lunch and dinner, suggesting it was the most fiber-efficient meal relative to energy consumed. A study reported that dinner accounted for the largest share of total daily energy intake (40%) among Japanese adults, which helps explain the lower fiber density observed for dinner in this study [17]. Snacks provided the lowest fiber (1.5 g) and density (7.8 g/1000 kcal) among all the meal types, consistent with previous reports indicating that snack consumption in Japan is largely composed of low-fiber food such as confectioneries and sugar-sweetened beverages [17]. These results suggest a need for practical strategies to motivate patients with T2D in Japan to increase fiber intake for breakfast and snacks, as well as to increase overall fiber density in all meals.

The absence of associations between fiber intake and participant characteristics such as age, sex, BMI, blood pressure, HbA_1c_, as well as meal logging frequency, suggests that these factors and the extent of meal self-monitoring may not strongly influence dietary fiber intake among patients with T2D in Japan. Other factors such as knowledge, attitude, culture, psychosocial factors, and socioeconomic status such as household income and education level may play a more prominent role in shaping dietary fiber intake behaviors in this population [19-22]. Future studies should investigate other behavioral and sociocultural determinants to better understand what drives dietary fiber intake and to inform the design of more effective interventions among patients with T2D in Japan.

### Strengths and Limitations

In this secondary analysis, 1 year of meal record data was used, providing a comprehensive assessment of dietary fiber intake that captured long-term eating habits and seasonal variations, as compared to assessments from short-term diet records.

We excluded days with incomplete meal records from the analysis, which may have favored inclusion of more engaged and health-motivated individuals. Participants who consistently logged all 3 meals may differ from those who did not, particularly in terms of health behaviors and motivation [23]. Therefore, the reported mean fiber intake of 17.1 g/day may overestimate the true average for the intervention users and population with T2D using similar app at large. Nevertheless, this method allowed uniform representation of dietary fiber intake assessment from each meal type and each participant and ensured that daily fiber estimates were not disproportionately influenced by partial or unbalanced records. It also provides insight into the dietary patterns of participants who were more adherent to meal logging as they are most likely to benefit from or respond to such digital dietary interventions. Moreover, the similarity in baseline characteristics between analyzed and excluded participants suggests that the analyzed sample remains representative of the broader study population, minimizing concerns about selection bias.

Due to dependence on self-reporting, reporting errors such as underreporting of food items and inaccurate portion size estimation may have occurred. We reduced the likelihood of reporting errors by using image-assisted food recording, which assisted participants in recording their meals accurately [24].

### Conclusions

Japanese patients with T2D who performed diet self-monitoring through the DialBetesPlus app consumed less than the recommended intake of 20 g and above of dietary fiber per day, on average over 1 year. Our findings suggest that even among highly motivated and digitally engaged participants, diet self-monitoring via the app alone did not lead to participants meeting the clinically recommended dietary fiber intake. This highlights the need to complement app-based tracking with additional evidence-based intervention features such as the use of fiber-focused intervention, goal setting, personalized feedback, action planning, and coping planning [25-29]. Exploring patients’ beliefs on and perceptions of dietary fiber intake is important to build user-informed dietary mHealth interventions [30].

## Supplementary material

10.2196/69340 Multimedia Appendix 1 Demographic and clinical characteristics of the total study population and the subgroup of participants excluded from analysis in a 12-month randomized controlled trial evaluating DialBetesPlus intervention for individuals with type 2 diabetes in Japan.

10.2196/69340 Multimedia Appendix 2 Comparison of baseline demographic and clinical characteristics between analyzed (N=47) and excluded (n=19) participants in a 12-month randomized controlled trial of the DialBetesPlus intervention for individuals with type 2 diabetes in Japan.

10.2196/69340 Multimedia Appendix 3 Q-Q plots evaluating the distribution of daily dietary fiber intake (S1A) and fiber density (S1B) among analyzed participants in a 12-month randomized controlled trial investigating DialbetesPlus intervention for individuals with type 2 diabetes in Japan.

10.2196/69340 Multimedia Appendix 4 Spearman correlation coefficients and corresponding *P* values between total dietary fiber intake and participant characteristics.

## Abbreviations

DBP: diastolic blood pressure
HbA_1c_: hemoglobin A_1c_
SBP: systolic blood pressure
T2D: type 2 diabetes
