# Supplementary material for: Dietary Fiber Estimate of DialBetesPlus App Users: Secondary Analysis of Data From a Randomized Controlled Trial
Source: JMIR Form Res. 2025 Oct 2;9:e69340. doi: 10.2196/69340 (PMC12490812; doi:10.2196/69340)
Supplement: Multimedia Appendix 1 [file formative-v9-e69340-s001.docx]

**Table S1.**

| Demographic characteristics | Excluded participants | | | Total study population | | |
| --- | --- | --- | --- | --- | --- | --- |
|  | Mean (SD) | Frequency (n) | n | Mean (SD) | Frequency (n) | N |
| Age | 54.6 (12.4) |  | 19 | 58.6 (10.1) |  | 66 |
| Sex  Male  Female |  | 34  13 | 19 |  | 47  19 | 66 |
| Baseline BMI | 30.0 (5.2) |  | 19 | 28.7 (5.3) |  | 66 |
| Changes in BMI post-intervention | -0.5 (1.2) |  | 15 | -0.7 (1.2) |  | 66 |
| Baseline HbA1c | 8.1 (1.8) |  | 15 | 7.8 (1.3) |  | 62 |
| Changes in HbA1c post-intervention | -0.5 (1.5) |  | 15 | -0.3 (1.0) |  | 62 |
| Baseline blood pressure (SBP/DBP, mmHg) | 130/80 (19/13) |  | 19 | 133/81 (16/11) |  | 66 |
| Changes in blood pressure (SBP/DBP, mmHg) | 11/1 (21/13) |  | 15 | 0/-2 (20/1) |  | 61 |
