# Supplementary material for: Dietary Fiber Estimate of DialBetesPlus App Users: Secondary Analysis of Data From a Randomized Controlled Trial
Source: JMIR Form Res. 2025 Oct 2;9:e69340. doi: 10.2196/69340 (PMC12490812; doi:10.2196/69340)
Supplement: Multimedia Appendix 2 [file formative-v9-e69340-s002.docx]

**Table S1.**

| Participant baseline characteristics | Test Used | Statistic | p-value |
| --- | --- | --- | --- |
| Age | Welch’s t-test | 1.798 | 0.0842 |
| Baseline BMI | Mann–Whitney U | 328.0 | 0.0947 |
| Baseline SBP | Welch’s t-test | 1.034 | 0.3099 |
| Baseline DBP | Welch’s t-test | 0.343 | 0.7338 |
| Baseline HbA1c | Mann–Whitney U | 350.0 | 0.9737 |
| Sex | Chi-squared test | 0.0 | 0.9855 |
