# Supplementary figures and images for: Dietary Fiber Estimate of DialBetesPlus App Users: Secondary Analysis of Data From a Randomized Controlled Trial
Source: JMIR Form Res. 2025 Oct 2;9:e69340. doi: 10.2196/69340 (PMC12490812; doi:10.2196/69340)

**Figure S1(A).**


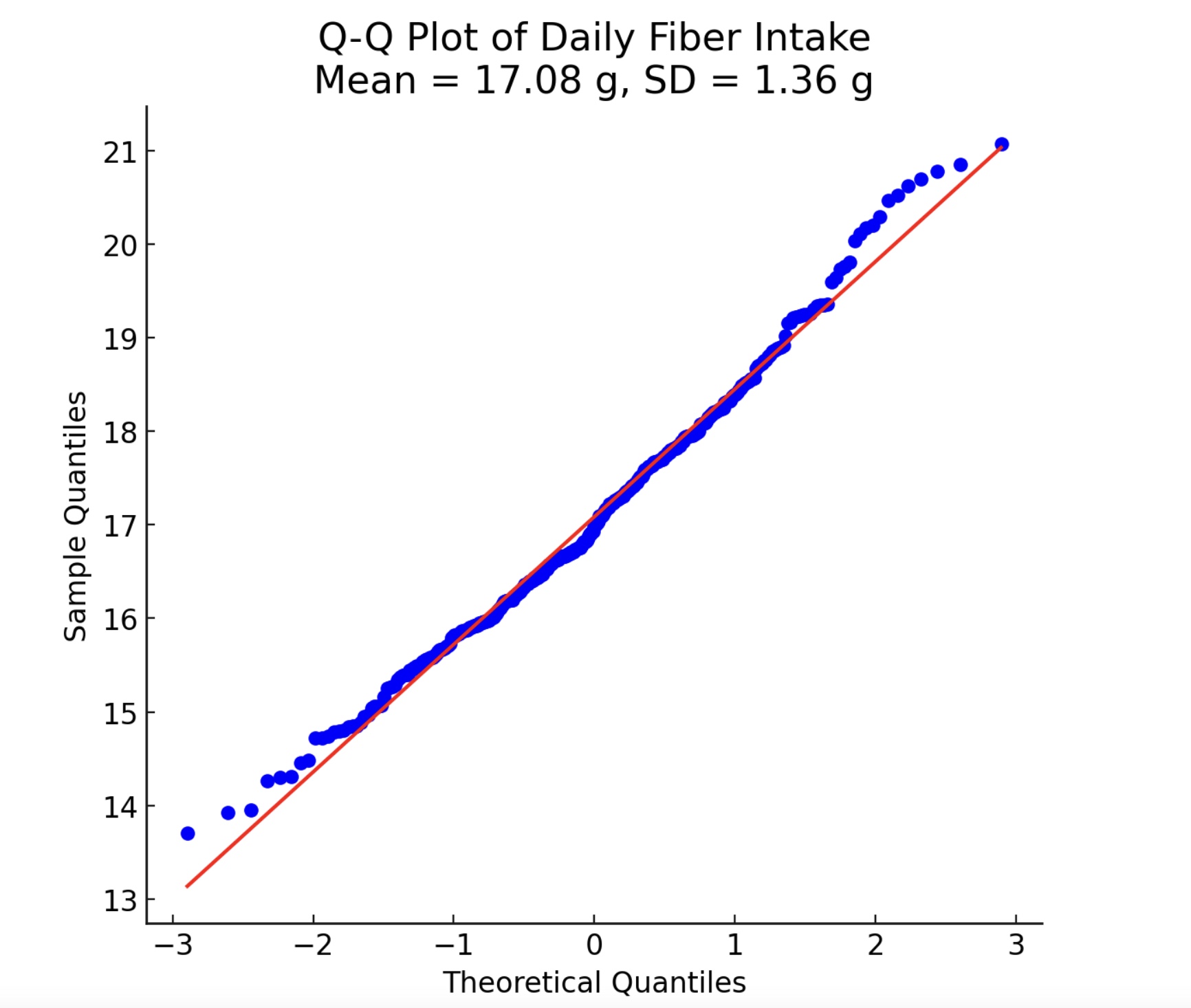


**Figure S1(B).**


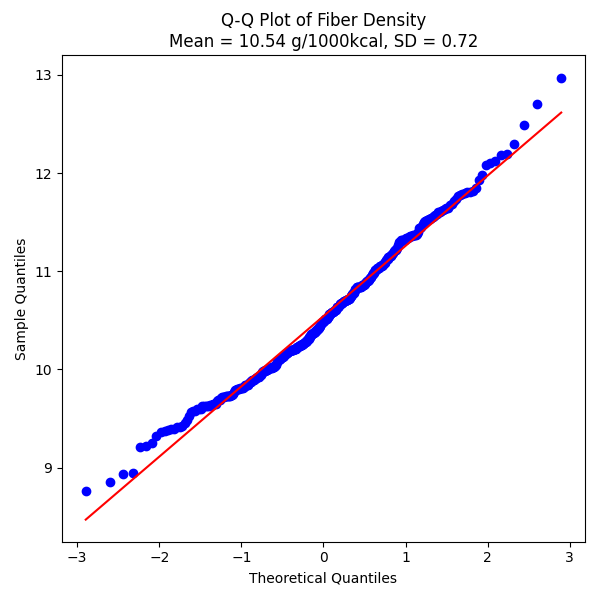

Supplement: Multimedia Appendix 3 [file formative-v9-e69340-s003.docx]
