# Supplementary material for: Dietary Fiber Estimate of DialBetesPlus App Users: Secondary Analysis of Data From a Randomized Controlled Trial
Source: JMIR Form Res. 2025 Oct 2;9:e69340. doi: 10.2196/69340 (PMC12490812; doi:10.2196/69340)
Supplement: Multimedia Appendix 4 [file formative-v9-e69340-s004.docx]

**Table S1.**

| Variable | Spearman rho | p-value |
| --- | --- | --- |
| Age | -0.02 | 0.893 |
| Sex | 0.072 | 0.6234 |
| Baseline BMI | 0.221 | 0.1268 |
| BMI change post intervention | 0.059 | 0.6896 |
| Baseline HbA1c | 0.049 | 0.7405 |
| HbA1c change post intervention | -0.015 | 0.9162 |
| Baseline SBP | 0.175 | 0.2285 |
| Baseline BBP | 0.015 | 0.919 |
| SBP change post intervention | -0.039 | 0.7905 |
| DBP change post intervention | -0.098 | 0.5085 |
